# Complement receptor type 1 and 2 (*CR1* and *CR2*) gene polymorphisms and plasma protein levels are associated with the Dengue disease severity

**DOI:** 10.1038/s41598-023-44512-w

**Published:** 2023-10-13

**Authors:** Nguy Thi Diep, Ngo Truong Giang, Nguyen Thi Thuy Diu, Nguyen Minh Nam, Le Van Khanh, Ha Van Quang, Ngo Thu Hang, Can Van Mao, Ho Van Son, Nguyen Lan Hieu, Phan Tung Linh, Ella H. Sklan, Nguyen Linh Toan, Hoang Van Tong

**Affiliations:** 1 Department of Pathophysiology, Vietnam Military Medical University, Hanoi, Vietnam; 2 Hanoi Nephrology Hospital, Hanoi, Vietnam; 3 Department of Biology and Medical Genetics, Vietnam Military Medical University, Hanoi, Vietnam; 4 Institute of Biomedicine and Pharmacy, Vietnam Military Medical University, 222 Phung Hung, Ha Dong, Hanoi, Vietnam; 5 103 Military Hospital, Vietnam Military Medical University, Hanoi, Vietnam; 6 175 Military Hospital, Ho Cho Minh City, Vietnam; 7 Hanoi Medical University Hospital, Hanoi Medical University, Hanoi, Vietnam; 8 Department of Clinical Microbiology and Immunology, Sackler Faculty of Medicine, Tel-Aviv University, 69978 Tel Aviv, Israel

**Keywords:** Complement cascade, Viral infection, Immunogenetics

## Abstract

The pathological outcome of dengue disease results from complex interactions between dengue virus (DENV) and host genetics and immune response. Complement receptor types 1 and 2 (CR1 and CR2) mediate complement activation through the alternative pathway. This study investigated the possible association of genetic polymorphisms and plasma levels of CR1 and CR2 with dengue disease. A total of 267 dengue patients and 133 healthy controls were recruited for this study. *CR1* and *CR2* gene polymorphisms were analyzed by Sanger sequencing, while plasma CR1 and CR2 levels were measured by ELISA. The frequency of the *CR1* minor allele *rs6691117G* was lower in dengue patients and those with severe dengue compared to healthy controls. Plasma CR1 and CR2 levels were decreased in dengue patients compared to healthy controls (*P* < 0.0001) and were associated with platelet counts. CR1 levels were lower in dengue patients with warning signs (DWS) compared to those without DWS, while CR2 levels were decreased according to the severity of the disease and after 5 days (T1) and 8 days (T2) of follow-up. CR2 levels were decreased in dengue patients positive for anti-DENV IgG and IgM and patients with bleeding and could discriminate DWS and SD from dengue fever patients (AUC = 0.66). In conclusion, this study revealed a reduction in CR2 levels in dengue patients and that the *CR1* SNP rs6691117A/G is associated with the dengue severity. The correlation of CR2 levels with platelet counts suggests that CR2 could be an additional biomarker for the prognosis of severe dengue disease.

## Introduction

Dengue fever (DF), caused by Dengue virus (DENV), is an acute infectious disease that spreads rapidly through *Aedes* mosquito bites. It is estimated that the number of cases has increased more than 30 times globally in the past 50 years, and more than 50 per cent of the world's population in more than 100 countries live in high-risk areas^1^. DENV comprises four antigenically distinct serotypes, including DENV-1, DENV-2, DENV-3, and DENV-4^2,3^. Dengue fever presents with diverse clinical manifestations, progressing rapidly from mild to severe. This disease often has a sudden onset and progresses through three stages: the febrile phase, the critical phase, and the convalescent phase^4^. Following guidelines for clinical management of the World Health Organization (WHO) in 2009, dengue fever is categorized into three categories: dengue fever (DF), dengue fever with warning signs (DWS), and severe dengue (SD). The latter, characterized by fever, haemorrhage, plasma leakage, and the potential for hypovolemic shock, coagulopathy, as well as visceral and hepatocellular damage, represents the most severe form of the disease^4^.

The pathogenesis of dengue virus infection is attributed to the complex combination of various factors including the virus, host genetics and immune response. These encompass non-structural protein 1 (NS1) viral antigen, variation in the DENV genome, subgenomic RNA, antibody-dependent enhancement (ADE), memory cross-reactive T cells, anti-DENV NS1 antibodies, autoimmunity, and host genetic variants. The combined effect of the above-mentioned factors causes endothelial cell dysfunction, plasma leakage, coagulopathy, and thrombocytopenia, ultimately leading to severe dengue symptoms^5^. Immune responses play a crucial role in modulating the pathogenesis of dengue fever. A study showed elevated levels of circulating cytokines and chemokines (referred to as cytokinemia) associated with massive immune activation in dengue fever cases^6^. Moreover, the complement system, a key component of host defence, exerts a protective function by restricting viral replication. Paradoxically, if excessively activated, it can contribute to the development of more severe disease by intensifying the inflammatory response^7^. Most of these activities are mediated by the complement receptors (CRs), including CR1 (CD35), CR2 (CD21), CR3 (CD11b/CD18), and CR4 (CD11c/CD18), which interact with the activation fragments of central components within the complement system, such as C3b, iC3b, and C3d^8,9^.

Complement receptor type 1 (CR1 or CD35) is a membrane protein that regulates complement activation and the control of immune responses through binding to C3b/C4b opsonized foreign antigens. The molecular weight of CR1 varies, ranging from 160 to 250 kDa depending on the type^9^. The *CR1* gene, also known as the C3b/C4b or CD35 receptor (differentiated cluster 35), is located on the long arm of chromosome 1, arm 32 (1q32) of the immune regulatory gene complex. Within this region, genes encode membrane cofactor proteins, including CR1 and CR2, followed by the promoter of degradation and C4-binding protein, organized sequentially from the 5'-3' end^10^. Complement receptor type 2 (CR2, CD21), encoded by a 20-exon gene located on chromosome 1q32.2^11^, is a 145 kDa protein, which is a type 1 membrane-bound glycoprotein consisting of 15–16 complement control protein (CCP) regions. This receptor comprises a 24 amino acid-long transmembrane and a relatively short, 34-amino acid-long intracellular region^12^.

Genetic polymorphisms and the plasma levels of CR1 and CR2 have previously been associated with various viral infections and other diseases^10,13–17^. For instance, a prior study demonstrated that two SNPs, namely rs3811381 and rs2274567, within the *CR1* gene contributed to an increased risk of developing chronic hepatitis B virus (HBV) and HBV-related hepatocellular carcinoma (HCC)^17^. In the context of severe fatal Coronavirus disease 2019 (COVID-19), CR1 expression was found to be reduced among a cluster of heterozygotes for CR1 genetic variants^18^. Additionally, a separate study revealed that the rs3813946 polymorphism in the *CR2* gene is associated with an increased susceptibility to human immunodeficiency virus 1 (HIV-1) infection following vaccination with recombinant glycoprotein 120 in human volunteers^19^. Another study reported a higher number of white blood cells expressing CR1 and CR2 receptors in patients with dengue fever compared to healthy controls^20^. At present, the pathogenesis of dengue virus infection remains incompletely understood, particularly the role of CR1 and CR2 proteins, along with *CR1* and *CR2* polymorphisms, in dengue disease has yet to be investigated. This present study aims to investigate the potential associations of *CR1, CR2* polymorphisms, and plasma CR1 and CR2 protein levels with the severity of dengue fever in Vietnamese patients.

## Materials and methods

### Patients and controls

A total of 267 Vietnamese patients admitted to the hospital with the symptoms of DENV infection were recruited for this study. The hospitals where the patients were recruited included the 103 Military Hospital, Vietnam Military Medical University (VMMU), 175 Military Hospital, Central Hospital for Tropical Diseases, and Hanoi Medical University Hospital from 2020 to 2023. Dengue patients were diagnosed based on the guidelines for the diagnosis of dengue according to the WHO 2009^4^. Dengue patients were further classified into three subgroups based on the 2009 WHO dengue classification, including patients with dengue fever without warning signs (DF, n = 154), those with dengue fever with warning signs (DWS, n = 99), and those with severe dengue (SD, n = 14)^4^. Patients diagnosed with dengue fever were identified based on the following criteria: residing in or travelling to a dengue-endemic area, presenting with fever, and exhibiting at least two of the following symptoms: nausea or vomiting, rash, aches and pains, a positive tourniquet test, a low white blood cell count, and laboratory-confirmed dengue infection, which include positivity for DENV NS1 or anti-DENV specific IgM or DENV PCR tests. Patients with dengue warning signs (DWS) were classified as those who not only had dengue fever but also displayed any of the following warning signs: abdominal pain or tenderness, persistent vomiting, clinical fluid accumulation, mucosal bleeding, lethargy, restlessness, liver enlargement exceeding 2 cm, or an increase in hematocrit (HCT) levels concurrent with a rapid decrease in platelet count. Patients were categorized as having severe dengue (SD) if they exhibited severe plasma leakage, severe bleeding, and severe organ impairment^4^. Exclusion criteria were dengue patients with co-morbidities such as hepatitis, cirrhosis, cancer, or other causes of thrombocytopenia, affecting the study results. In the patient group without warning signs, 36 dengue patients were followed up, and their blood samples were collected at the time of hospital admission (T0), after 5 days of admission (T1), and after 8 days of admission (T2). Laboratory data such as anthropometric, epidemiological, biochemical, and haematological parameters and clinical symptoms of dengue patients were recorded. The detection of NS1 antigen, anti-DENV IgG and IgM antibodies were done by quick test followed the routine serological procedures of the hospitals.

For the control group, we recruited 133 individuals who visited VMMU and 175 Military Hospital from 2021 to 2023 for regular health checks. The control individuals had clinical examinations and tests for blood counts and biochemical parameters such as liver enzymes such as aspartate aminotransferase (AST), alanine transaminase (ALT), and gamma-glutamyl transferase (GGT), as well as levels of glucose, urea, creatinine, and bilirubin. These individuals were also confirmed negative for HBV and HIV infections. Blood samples were collected on the day of admission (for all dengue patients) and further time points at T1 and T2 (for the follow-up group) and on the day of the visit for a health check (for healthy controls). The plasma samples were separated from the blood, transferred to a fresh polypropylene tube, and stored at -80 °C until use.

### Ethics statement

All study participants were provided with thorough information about the study, and informed consent was given from all participants or parents/guardians if the participants were under 16. All methods applied in this study were performed according to relevant clinical guidelines and experimental regulations. The VMMU Ethics Board evaluated and approved all clinical procedures on date of November 13th, 2020 according to the Decision number 4940/QĐ-HVQY.

### Determination of CR1 and CR2 polymorphisms by Sanger sequencing

Genomic DNAs were extracted from whole blood samples using the Gene JET Whole Blood Genomic DNA Purification Kit (Thermo, USA). The exon 29 of the *CR1* gene was amplified using CR1F: 5’-TCT TCA TAA ATA ATG CCA GAA GTG G-3' and CR1R: 5’-TGC CAA TTT CAT AGT CCT TAT ACA C-3′^10^. The temperature cycling was as follows: 95 °C for 5 min, followed by 40 cycles of denaturalization at 95 °C for 30 s, annealing at 61 °C for 30 s and extension at 72 °C for 45 s, and final extension for 10 min at 72 °C. The total reaction mixture (25 µl) contained 12.5 µl DreamTaq Green PCR Master Mix (2X) (Thermo, USA), 1 µl (0.2 μM) of each primer, and 3 µl (5–15 ng) DNA template. The exon 10 of the *CR2* gene was amplified using forward primer CR2F: 5’-TAT GGA ACC ACG GTC ACT TAC-3' and reverse primer CR2R: 5’-GGC AGA AGG ACT CCA ATT TCC-3′, which were designed by Primer3 software. PCR amplification was conducted in a 25 μl volume containing 12.5 µl GoTaq Colorless Master Mix (Promega, USA), 1 µl (0.2 μM) of each primer, and 3 µl (5–15 ng) DNA template. Cycling conditions were denaturation at 95 °C for 5 min, followed by 40 cycles of three-step cycling with denaturation (94 °C, 30 s), annealing (63 °C, 30 s), and extension (72 °C, 30 s) and final extension (72 °C, 10 min).

PCR products were purified using the GeneJET PCR Purification Kit (Thermo, USA). Purified DNA products were subjected to Sanger sequencing in ABI3500 System (Applied Biosystems, USA). Sequencing data were analyzed using MegaX32 to determine *CR1* and *CR2* polymorphisms by assembling with the reference sequence of *CR1* (NG_007481.1) and *CR2* (NG_013006.1) genes obtained from the NCBI database.

### Quantification of plasma CR1 and CR2 levels by ELISA

The plasma CR1 and CR2 protein concentrations in the dengue patients and healthy individuals were quantified by Enzyme-linked Immunosorbent Assay (ELISA) using Thermo Fisher Scientific's Human CD35 ELISA kit (code: EH87RB) and Human CD21/CR2/EBV receptor ELISA kit (code: EH81RB), respectively, according to the manufacturer's instructions.

### Statistical analysis

The data were organized using Microsoft Office Excel-2010 software, and statistical analyses were performed using SPSS v.20.0 software (SPSS Statistics, IBM, Armonk, NY, USA). For quantitative variables, we tested for normality by the Kolmogorov–Smirnov test. Student's t and ANOVA tests were used to compare means between groups if the data were normally distributed. Mann–Whitney U and Kruskal–Wallis tests were used to compare means between groups if the data were not normally distributed. The genotype frequencies were determined by simple counting. All study *CR1* and *CR2* gene polymorphisms were tested for Hardy–Weinberg equilibrium. CR2 haplotypes were analyzed using the expectation-maximum (EM) algorithm implemented in the Arlequin v. 3.5.1.2 software. Chi-square or Fisher's exact tests were used to test the difference between variables. A binary logistic regression model adjusted for confounding factors (age and gender) was used to analyze the association between *CR1* and *CR2* gene polymorphisms and dengue fever based on genetic models, including allele, dominant and recessive phenotypes. Odds ratios (ORs) and 95% confidence intervals (CIs) were calculated. A linear regression model examined the correlation between CR1, CR2 levels, and clinical parameters. Statistical significance was set to a *P* value of ≤ 0.05.

## Results

### Demographic, clinical, and biochemical characteristics of the study participants

The demographic, clinical, and biochemical characteristics of dengue fever patients are summarized in Table 1. There was no statistical difference in mean age, gender ratio, red blood cell (RBC) count, rate positivity of DENV NS1, and anti-DENV IgM between DF and DWS patients as well as between DF and SD patients (*P* > 0.05). However, the positivity rate of anti-DENV-IgG was higher in SD patients compared with those with DF (*P* = 0.01). The average number of days of onset at the time of sampling ranged from 3–4 days, and there was a difference between DF and DWS groups (*P* < 0.001). We observed that 51 out of 99 (51.5%) DWS patients and 4 out of 14 (28.6%) had internal or mucosal bleeding, whereas these symptoms were not observed in DF patients. The levels of AST and ALT were highest in the severe dengue group and gradually decreased in the DWS and dengue fever groups (*P* < 0.001). The platelet (PLT) count decreased in all disease groups, with the most substantial decrease recorded in the severe dengue and DWS groups compared to DF patients (*P* < 0.001). In addition, we observed statistical differences in the white blood cell (WBC) count in DWS and SD patients compared to DF patients, while hematocrits (HCT) were higher in DWS compared to DF patients (*P* = 0.004) (Table 1).

**Table 1:** Main characteristics of dengue patients.

Characteristics	DF (n = 154)	DWS (n = 99)	SD (n = 14)	*P* value (DF vs. DWS)	*P* value (DF vs. SD)
Age (years)	33 (8–79)	36 (15–81)	40.5 (16–82)	> 0.05*	> 0.05*
Gender				> 0.05**	> 0.05**
Male n (%)	93 (60.4)	55 (55.6)	5 (35.7)		
Female n (%)	61 (39.6)	44 (44.4)	9 (64.3)		
Number of illness days	3 (1–10)	4 (1–7)	3.5 (2–8)	< 0.001*	> 0.05*
Internal or mucosal bleeding					
Yes n (%)	0 (0)	51 (51.5)	4 (28.6)	< 0.001**	< 0.001**
No n (%)	154 (100)	48 (48.5)	10 (71.4)		
Liver enzyme					
AST (IU/ml)	51.5 (14–994)	112 (20–1564)	764.15 (274–7962)	< 0.001*	< 0.001*
ALT (IU/ml)	37 (7.3–589.81)	65.72 (12–9107)	208.85 (39–1769)	< 0.001*	< 0.001*
Blood counts					
RBC (× 10^3^/ml)	4.92 (3.49–7.11)	5.03 (3.71–6.34)	4.67 (1.56–7.47)	> 0.05*	> 0.05*
WBC (× 10^6^/ml)	4.2 (1–35.4)	3.6 (1–14.3)	5.8 (2.54–11.8)	0.029*	0.046*
PLT (× 10^3^/ml)	121.5 (5–343)	38 (3–309)	26 (5–171)	< 0.001*	< 0.001*
HCT (%)	42.56 ± 4.96	44.34 ± 4.57	37.36 ± 10.77	0.004***	> 0.05***
DENV NS1					
Positive n (%)	106 (68.8)	78 (78.8)	10 (71.7)	> 0.05**	> 0.05**
Negative n (%)	18 (11.7)	9 (9.1)	2 (14.3)		
Undetermined n (%)	30 (19.5)	12 (12.1)	2 (14.3)	NA	NA
Anti-DENV IgM					
Positive n (%)	63 (40.9)	40 (40.4)	10 (71.7)	> 0.05**	> 0.05**
Negative n (%)	54 (35.1)	35 (35.4)	2 (14.3)		
Undetermined n (%)	37 (24)	24 (24.2)	2 (14.3)	NA	NA
Anti-DENV IgG					
Positive n (%)	31 (20.1)	34 (34.3)	8 (57.1)	> 0.05**	0.01**
Negative n (%)	45 (29.2)	28 (28.3)	1 (7.1)		
Undetermined n (%)	78 (50.6)	37 (37.4)	5 (35.7)	NA	NA

*DENV* dengue virus, *DF* Dengue fever, *DWS* dengue fever with warning signs, *SD* severe dengue, *AST* aspartate aminotransferase, *ALT* alanine aminotransferase, *WBC* white blood cells, *RBC* red blood cells, *PLT* platelets, *HCT* Hematocrits, *IU* international unit, *NA* not applicable.

(*) *P* values were calculated by the Mann–Whitney *U* test (*), Chi-square or Fisher’s exact tests (**) and Student's t-test (***).

### Association of CR1 and CR2 gene polymorphism with dengue fever

The genotype and allele frequencies of the *CR1* SNP (*rs669117A/G*) and four *CR2* SNPs (*rs1048971G/A*, *rs17615G/A*, *rs4308977T/C*, *rs17616G/A)* determined in 267 patients with dengue fever and 133 healthy individuals are presented in Tables 2 and 3. The analyzed SNPs in healthy controls adhered to Hardy–Weinberg equilibrium (*P* > 0.05). We proceeded to compare the genotype and allele frequencies between dengue fever patients and healthy individuals. In the *CR1* gene, we observed that the frequency of heterozygous genotype *rs6691117GA* and homozygous genotype *rs6691117AA* appeared with relatively high frequency (> 25%) in all groups. The frequency of the minor allele *rs6691117G* was significantly lower in dengue patients and in those with severe dengue compared to those in the healthy controls (OR = 0.73, 95% CI 0.54–0.99, *P* = 0.045; OR = 0.21, 95% CI 0.07–0.63, *P* = 0.005) suggesting that the *CR1* SNP rs6691117A/G are associated with protection from dengue fever. The frequency of minor allele *rs6691117G* was also significantly lower in the severe dengue group in comparison to those in patients with dengue fever and DWS (dengue fever: OR = 0.31, 95% CI 0.11–0.94, *P* = 0.038; DWS: OR = 0.27, 95% CI 0.09–0.81, *P* = 0.02) suggesting a protective effect against disease progression to the severe stage. Furthermore, we observed a similar trend of protective effect for the *CR1* SNP rs6691117A/G in the dominant genetic model (Table 2).

**Table 2:** Association of *CR1* polymorphisms with dengue fever in Vietnamese patients.

*CR1* SNP	All Patients	DF	DWS	SD	Healthy Controls	All Patients vs. Healthy Controls		SD vs. Healthy Controls		DF vs. SD		DWS vs. SD	
	n = 267 (%)	n = 154 (%)	n = 99 (%)	n = 14 (%)	n = 133 (%)	OR (95% CI)	*P* value	OR (95% CI)	*P* value	OR (95% CI)	*P* value	OR (95% CI)	*P* value
rs6691117 (A/G)													
* AA*	111 (41.6)	63 (40.9)	38 (38.4)	10 (71.4)	44 (33.1)								
* GA*	124 (46.4)	67 (43.5)	53 (53.5)	4 (28.6)	65 (48.9)								
* GG*	32 (12)	24 (15.6)	8 (8.1)	0 (0)	24 (18)								
* P* for H&W equilibrium	0.769	0.383	0.072	0.533	0.999								
Allelic													
* A*	346 (64.8)	193 (63.7)	129 (65.2)	24 (85.7)	153 (57.5)		Ref		Ref		Ref		Ref
* G*	188 (35.2)	115 (37.3)	69 (34.8)	4 (14.3)	113 (42.5)	**0.73 (0.54–0.99)**	**0.045**	**0.21 (0.07–0.63)**	**0.005**	**0.31 (0.11–0.94)**	**0.038**	**0.27 (0.09–0.81)**	**0.02**
Dominant													
* AA*	111 (41.6)	63 (40.9)	38 (38.4)	10 (71.4)	44 (33.1)		Ref		Ref		Ref		Ref
* GA* + *GG*	156 (58.4)	91 (59.1)	61 (61.6)	4 (28.6)	89 (66.9)		NS	**0.19 (0.06–0.64)**	**0.007**	0.31 (0.09–1.06)	0.062	**0.19 (0.05–0.72)**	**0.014**
Recessive							NS		NS		NS		NS
* AA* + *AG*	235 (88)	130 (84.4)	91 (91.9)	14 (100)	109 (82)		Ref		Ref		Ref		Ref
* GG*	32 (12)	24 (15.6)	8 (8.1)	0	24 (18)		NS		NA		NA		NA

Significant values are in [bold].

*DF* Dengue fever, *DWS* dengue fever with warning signs, *SD* severe dengue, *H&W* Hardy–Weinberg equilibrium, *NA* not applicable, *NS* not significant, *Ref* Reference.

*P* values were calculated by using a binary logistic regression model and adjusted for age and gender.

**Table 3:** Genotype and allele distribution of *CR2* polymorphisms in dengue fever patients and healthy controls.

CR2 position	All Patients	DF	DWS	SD	Healthy Controls	SD vs. Healthy Controls		DF vs. SD		SD vs. DWS	
rs1048971 (G/A)	n = 267 (%)	n = 154 (%)	n = 99 (%)	n = 14 (%)	n = 133 (%)	OR (95% CI)	*P* value	OR (95% CI)	*P* value	OR (95% CI)	*P* value
*GG*	175 (65.5)	102 (66.2)	67 (67.7)	6 (42.9)	84 (63.2)		Ref		Ref		Ref
*GA*	75 (28.1)	43 (27.9)	26 (26.3)	6 (42.9)	44 (33.1)		NS		NS		NS
*AA*	17 (6.4)	9 (5.8)	6 (6.1)	2 (14.3)	5 (3.8)	**3.07 (1.08–8.71)**	**0.035**		NS		NS
*P* for H&W equilibrium	0.026	0.133	0.127	0.8	0.796						
*G*	425 (79.6)	247 (80.2)	160 (80.1)	18 (64.3)	212 (79.7)		Ref		Ref		Ref
*A*	109 (20.4)	61 (19.8)	38 (19.9)	10 (35.7)	54 (20.3)	2.17 (0.95–4.99)	0.068	2.08 (0.9–4.81)	0.088	2.28 (0.96–5.41)	0.062

**rs17615 (G/A)**	**n = 267 (%)**	**n = 154 (%)**	**n = 99 (%)**	**n = 14 (%)**	**n = 133 (%)**						
*GG*	202 (75.7)	120 (77.9)	73 (73.7)	9 (64.3)	99 (74.4)		Ref		Ref		Ref
*GA*	59 (22.1)	31 (20.1)	24 (24.2)	4 (28.6)	31 (23.3)		NS		NS		NS
*AA*	6 (2.2)	3 (1.9)	2 (2.0)	1 (7.1)	3 (2.3)		NS		NS		NS
*P* for H&W equilibrium	0.499	0.553	0.987	0.57	0.757						
*G*	463 (86.7)	271 (88)	170 (85.9)	22 (78.6)	229 (86.1)						
*A*	71 (13.2)	37 (12)	28 (14.1)	6 (21.1)	37 (13.9)						

**rs4308977 (T/C)**	**n = 267 (%)**	**n = 154 (%)**	**n = 99 (%)**	**n = 14 (%)**	**n = 133 (%)**						
*TT*	202 (75.7)	120 (77.9)	73 (73.7)	9 (64.3)	100 (75.2)		Ref		Ref		Ref
*TC*	59 (22.1)	31 (20.1)	24 (24.2)	4 (28.6)	30 (22.6)		NS		NS		NS
*CC*	6 (2.2)	3 (1.9)	2 (2.0)	1 (7.1)	3 (2.3)		NS		NS		NS
*P* for H&W equilibrium	0.499	0.553	0.987	0.57	0.676						
*T*	463 (86.7)	271 (88)	170 (85.9)	22 (78.6)	230 (86.5)		Ref		Ref		Ref
*C*	71 (13.2)	37 (12)	28 (14.1)	6 (21.1)	36 (13.5)		NS		NS		NS

**rs17616 (G/A)**	**n = 267 (%)**	**n = 154 (%)**	**n = 99 (%)**	**n = 14 (%)**	**n = 133 (%)**						
GG	201 (75.3)	120 (77.9)	72 (72.7)	9 (64.3)	99 (74.4)		Ref		Ref		Ref
GA	59 (22.1)	31 (20.1)	24 (24.2)	4 (28.6)	31 (23.3)		NS		NS		NS
AA	7 (2.6)	3 (1.9)	3 (3.0)	1 (7.1)	3 (2.3)		NS		NS		NS
*P* for H&W equilibrium	0.297	0.553	0.57	0.57	0.757						
*G*	461 (86.3)	271 (88)	168 (84.8)	22 (78.6)	229 (86.1)		Ref		Ref		Ref
*A*	73 (13.7)	37 (12)	30 (15.2)	6 (21.1)	37 (13.9)		NS		NS		NS

*DF* Dengue fever, *DWS* dengue fever with warning signs, *SD* severe dengue, *H&W* Hardy–Weinberg equilibrium, *NA* not applicable, *NS* not significant, *Ref.* Reference.

*P* values were calculated by using a binary logistic regression model and adjusted for age and gender.

In the *CR2* gene, the frequency of minor allele *rs1048971A* was found to be significantly higher in patients with severe dengue compared to those in patients with dengue fever (OR = 2.2, 95% CI 0.9–5.4, *P* = 0.048) and those in patients with DWS (OR = 2.3, 95% CI 0.9–5.8, *P* = 0.045) suggesting that the *CR2* SNP *rs1048971G/A* are associated with an increased risk of progressing to severe dengue (Supplementary table 4). However, the difference did not reach statistical significance when adjusted for age and gender in either a logistic regression model or in dominant and recessive genetic models. We did not observe any significant difference in all comparisons of other *CR2* SNPs (rs17615G/A, rs4308977T/C, rs17616G/A) (Table 3). Haplotypes were constructed based on the four *CR2* SNPs (rs1048971G/A, rs17615G/A, rs4308977T/C, rs17616G/A), and the frequencies were presented in Supplementary Table 5. We identified three haplotypes having a relatively high frequency (> 5%), *GGTG, AGTG, AACA*, and several minor haplotype types (*GGTA, AGTT, GGTT, AATA*) with relatively low frequencies (< 4%). However, no significant difference in haplotype frequencies between groups was observed (*P* > 0.05) (Supplementary table 5).

### CR1 and CR2 protein levels in dengue patients and healthy individuals

**Figure 1:**
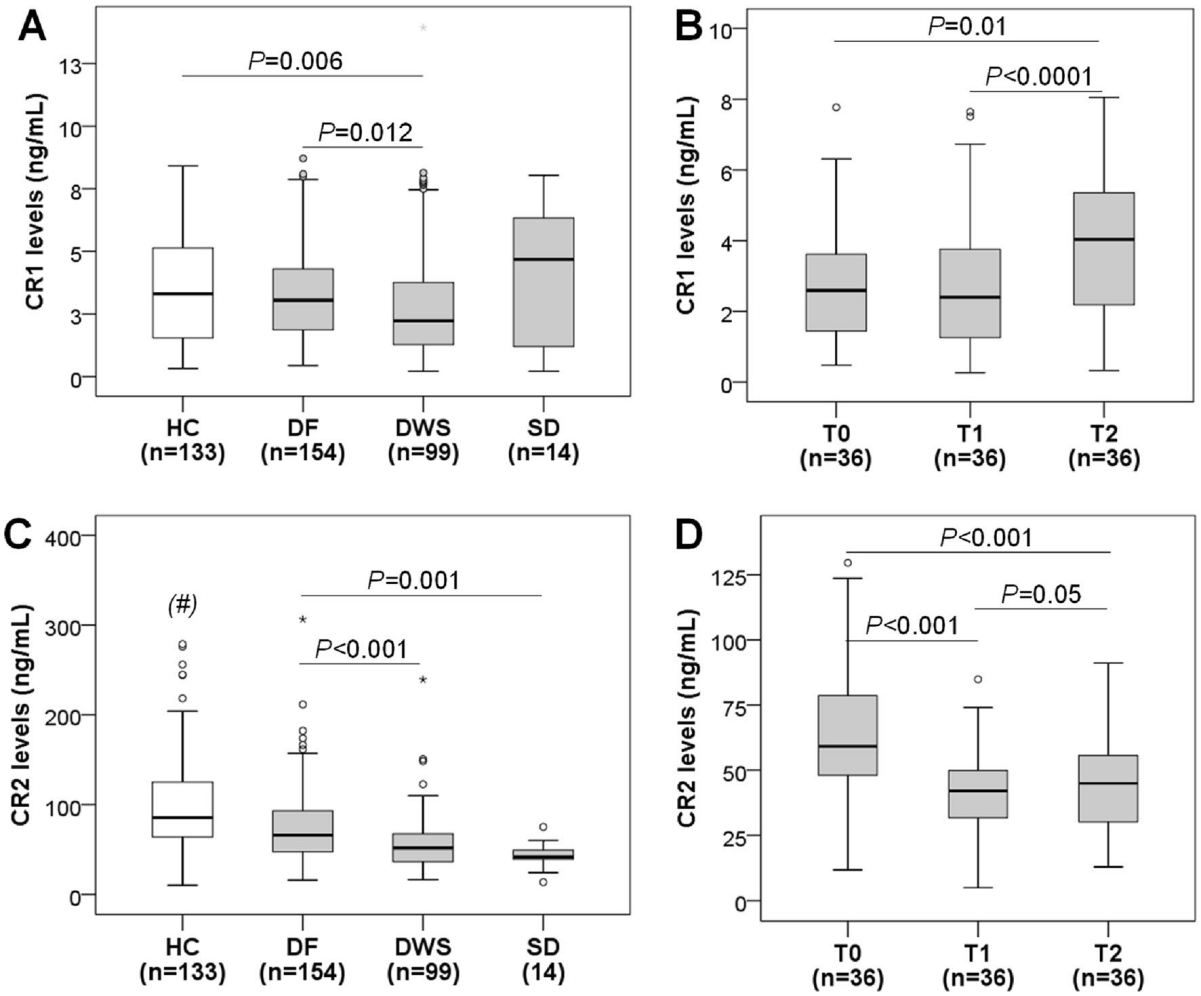
CR1 and CR2 levels in dengue patients and healthy individuals. (**A**): CR1 levels in different groups of dengue patients and healthy controls. (**B**): CR1 levels in dengue patients with follow-up at the time of admission (T0), after 5 days of admission (T1), and after 8 days of admission (T2). (**C**): CR2 levels in different groups of dengue patients and healthy controls. (**D**): CR2 levels in dengue patients with follow-up at the time of admission (T0), after 5 days of admission (T1), and after 8 days of admission (T2). Box plots present medians with 25 and 75 percentiles with whiskers to 10 and 90 percentiles, the dots and asterisks are the outliners; HC: healthy control; DF: Dengue fever; DWS: dengue patients with warning signs. *P* values were calculated by the Mann–Whitney U test. (#): *P* < 0.0001 compared with DF, DWS and SD groups.

CR1 and CR2 protein levels were quantified in the plasma of the study subjects and compared between various groups. The study findings revealed a decrease in CR1 levels in patients with DWS compared to healthy controls (*P* = 0.006). CR1 levels were lower in patients with DWS than in DF patients (*P* = 0.012). Notably, we observed the highest levels of CR1 in patients with severe dengue compared to other groups. However, this difference did not reach statistical significance (Fig. 1A). We further compared CR1 protein levels in the followed-up dengue patients at different time points, on the day of admission (T0), after 5 days of admission (T1), and after 8 days of admission (T2). The results revealed a slight decrease in CR1 levels after 5 days of admission (T1) compared to T0, followed by a significant elevation after 8 days of admission (T2) (*P* < 0.0001) (Fig. 1B).

Regarding the CR2 protein, we also observed that CR2 levels were significantly decreased in all dengue patient groups when compared to healthy controls (*P* < 0.0001). Within the dengue patient groups, the CR2 protein levels were lowest in severe dengue patients, followed by patients with DWS and dengue fever patients (*P* < 0.001). This observation suggests a notable reduction in CR2 levels corresponding to the severity of the disease (Fig. 1C). Furthermore, we observed a decrease in CR2 levels after 5 days (T1) and 8 days (T2) of follow-up in comparison to levels on the day of admission (T0) (*P* < 0.001) (Fig. 1D).

**Figure 2:**
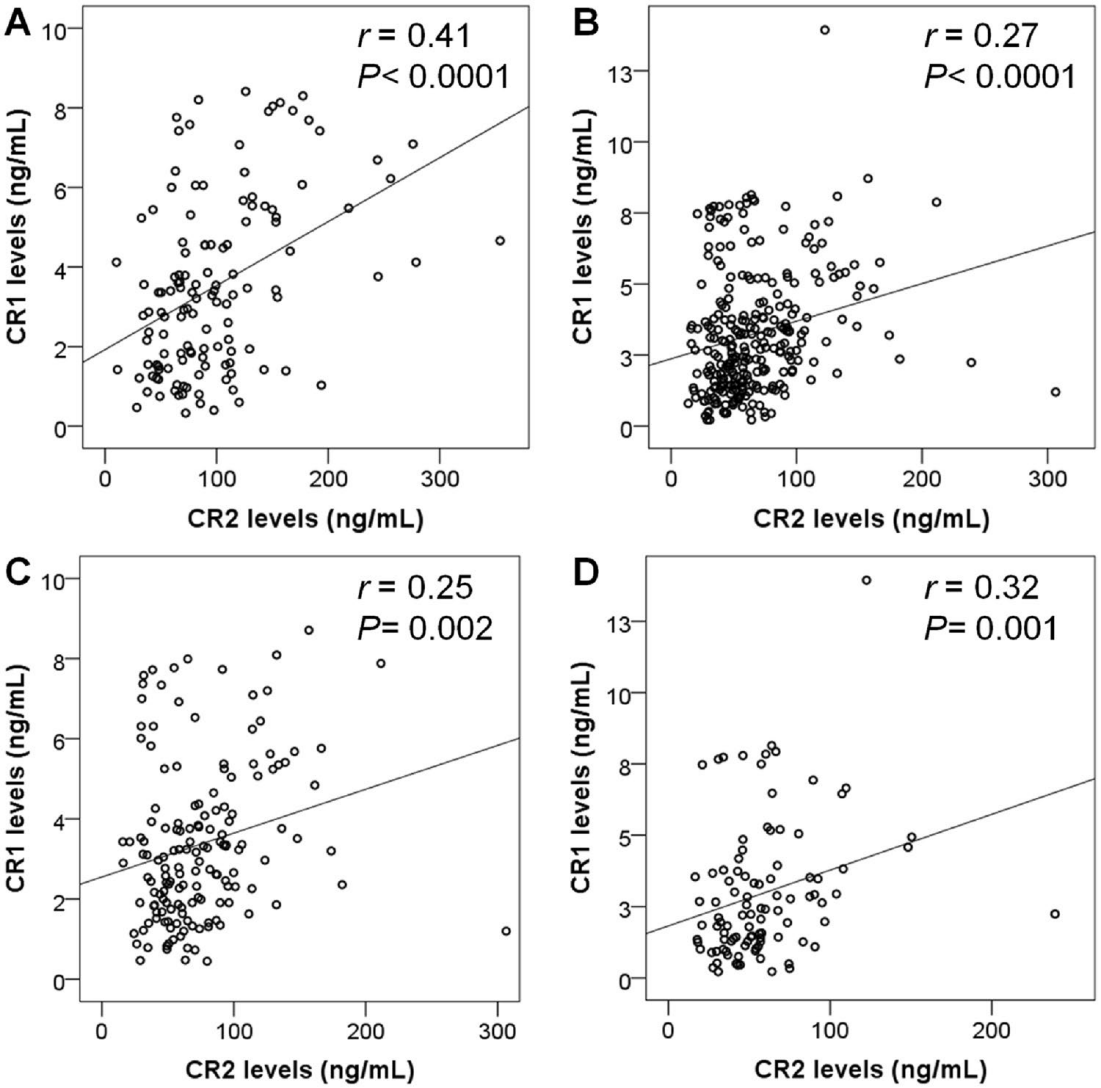
Correlation of CR1 and CR2 protein levels in healthy controls and dengue patients. (**A**) in healthy controls; (**B**): in all dengue patients; (**C**): in patients with dengue fever; (**D**) in patients with dengue fever with warning signs. rho (ρ) and *P* values were calculated by Spearman's correlation.

In addition, we also analyzed the correlation between CR1 and CR2 levels in healthy controls and dengue patients. The results revealed that CR1 levels were positively correlated with CR2 levels in both healthy individuals and dengue patients (Spearman's rho = 0.41 and 0.27, respectively, *P* < 0.0001). CR1 levels were positively correlated with CR2 levels in DF patients (Spearman's rho = 0.25, *P* = 0.002) and in dengue patients with warning signs (Spearman's rho = 0.32, *P* = 0.001) (Fig. 2).

### Association of CR1 and CR2 levels with dengue tests

**Figure 3:**
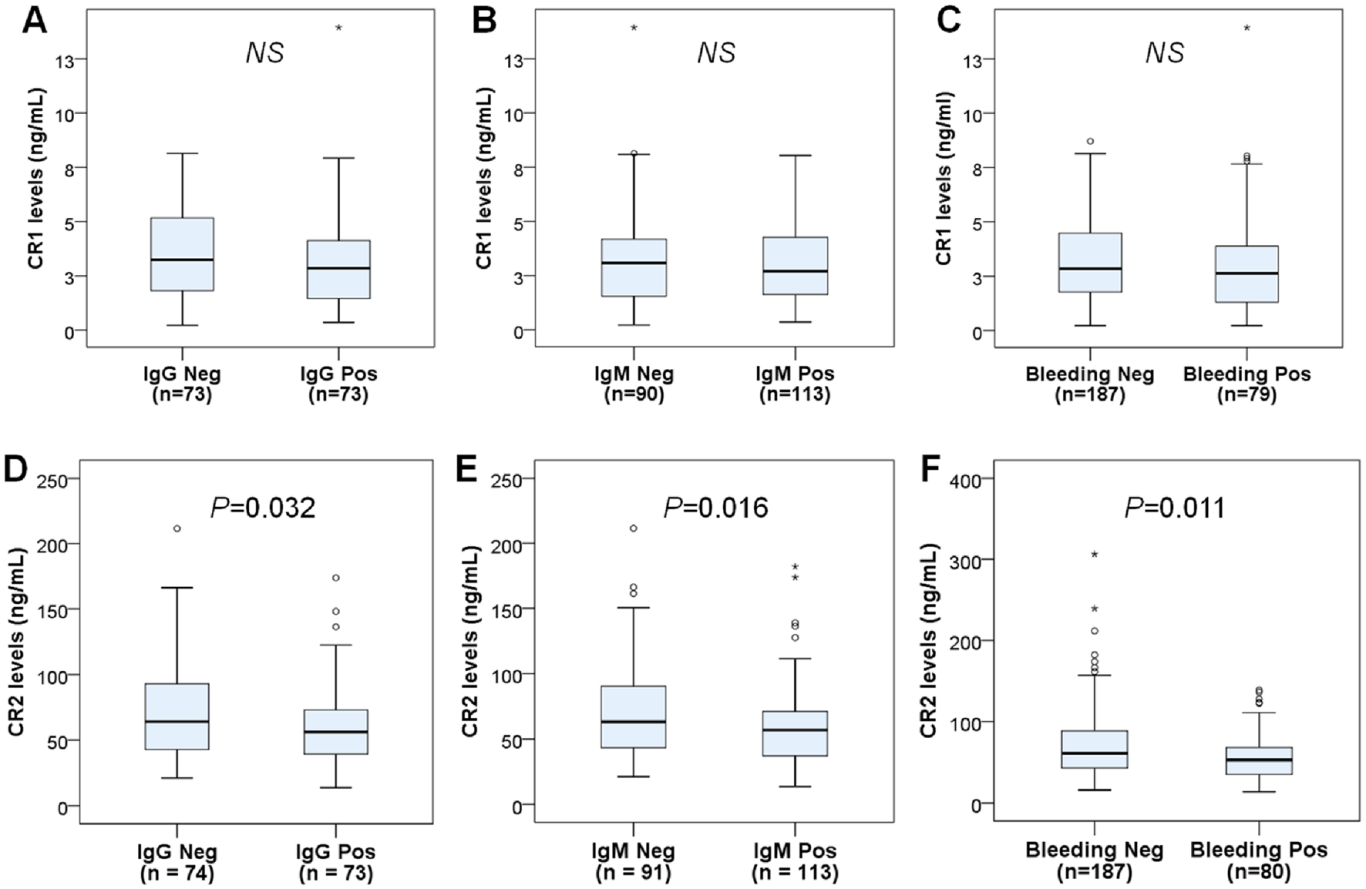
CR1 and CR2 levels in dengue patients according to dengue tests. (**A**): CR1 levels in dengue patients with negative and positive anti-DENV specific IgG. (**B**): CR1 levels in dengue patients with negative and positive anti-DENV specific IgM. (**C**): CR1 levels in dengue patients with negative and positive bleeding tests. (**D**): CR2 levels in dengue patients with negative and positive anti-DENV specific IgG. (**E**): CR2 levels in dengue patients with negative and positive anti-DENV specific IgM. (**F**): CR2 levels in dengue patients with negative and positive bleeding tests. Box plots present medians with 25 and 75 percentiles with whiskers to 10 and 90 percentiles, the dots and asterisks are the outliners; Neg: negative; Pos: positive; NS: not significant. *P* values were calculated by the Mann–Whitney *U* test.

In dengue patients, we segregated them based on serological test results into subgroups, including patients with anti-DENV specific IgG positive and negative, those with anti-DENV specific IgM positive and negative, and those with and without internal/mucosal bleeding, and compared CR1 and CR2 levels between subgroups. The results revealed that CR2 levels were decreased in dengue patients positive for anti-DENV specific IgG and IgM and internal/mucosal bleeding (*P* = 0.032, 0.016, and 0.011, respectively) (Fig. 3). A similar trend was observed for CR1. However, the difference was not statistically significant. These results suggest that previous and current infections with DENV contribute to modulating plasma CR1 and CR2 levels.

### Correlation of CR1 and CR2 levels with clinical parameters

We analyzed the correlation of the plasma CR1 and CR2 levels with several laboratory parameters in both healthy controls and dengue patients. Among dengue patients, we observed that CR1 levels were positively correlated with platelet counts (Spearman's rho = 0.23; *P* < 0.0001) and weakly correlated with white blood cells (Spearman's rho = 0.16; *P* = 0.01), while CR2 levels were positively correlated with platelet counts (Spearman's rho = 0.33; *P* < 0.0001) and inversely correlated with AST (Spearman's rho = − 0.23; *P* < 0.0001). Among healthy controls, CR2 levels were weakly correlated with ALT (Spearman's rho = -0.18; *P* = 0.041) (Table 4). These findings suggest that CR1 and CR2 levels could serve as an additional indicator of DENV infection.

**Table 4:** Correlation between CR1, CR2 serum levels and paraclinical indicators in dengue fever patients.

Parameter		WBC (× 10^3^/ml)	PLT (× 10^3^/mL)	AST (IU/mL)	ALT (IU/mL)
All dengue patients					
CR1 (ng/mL)	ρ (rho)	0.16	**0.23**	− 0.04	0.02
	P value	0.01	** < 0.0001**	0.5	0.75
CR2 (ng/mL)	ρ (rho)	− 0.005	**0.33**	**− 0.23**	− 0.16
	P value	0.94	** < 0.0001**	** < 0.0001**	0.009
Healthy controls					
CR1 (ng/mL)	ρ (rho)	− 0.02	− 0.062	− 0.17	− 0.11
	P value	0.99	0.47	0.056	0.19
CR2 (ng/mL)	ρ (rho)	− 0.057	0.001	− 0.15	**− 0.18**
	P value	0.52	0.99	0.091	**0.041**

*AST* aspartate aminotransferase, *ALT* alanine aminotransferase, *WBC* white blood cells, *PLT* platelets, *IU* international unit.

rho (ρ) and *P* values were calculated using Spearman's correlation. Significant values are in bold.

### Prognostic significance of CR1 and CR2 levels for dengue fever

**Figure 4:**
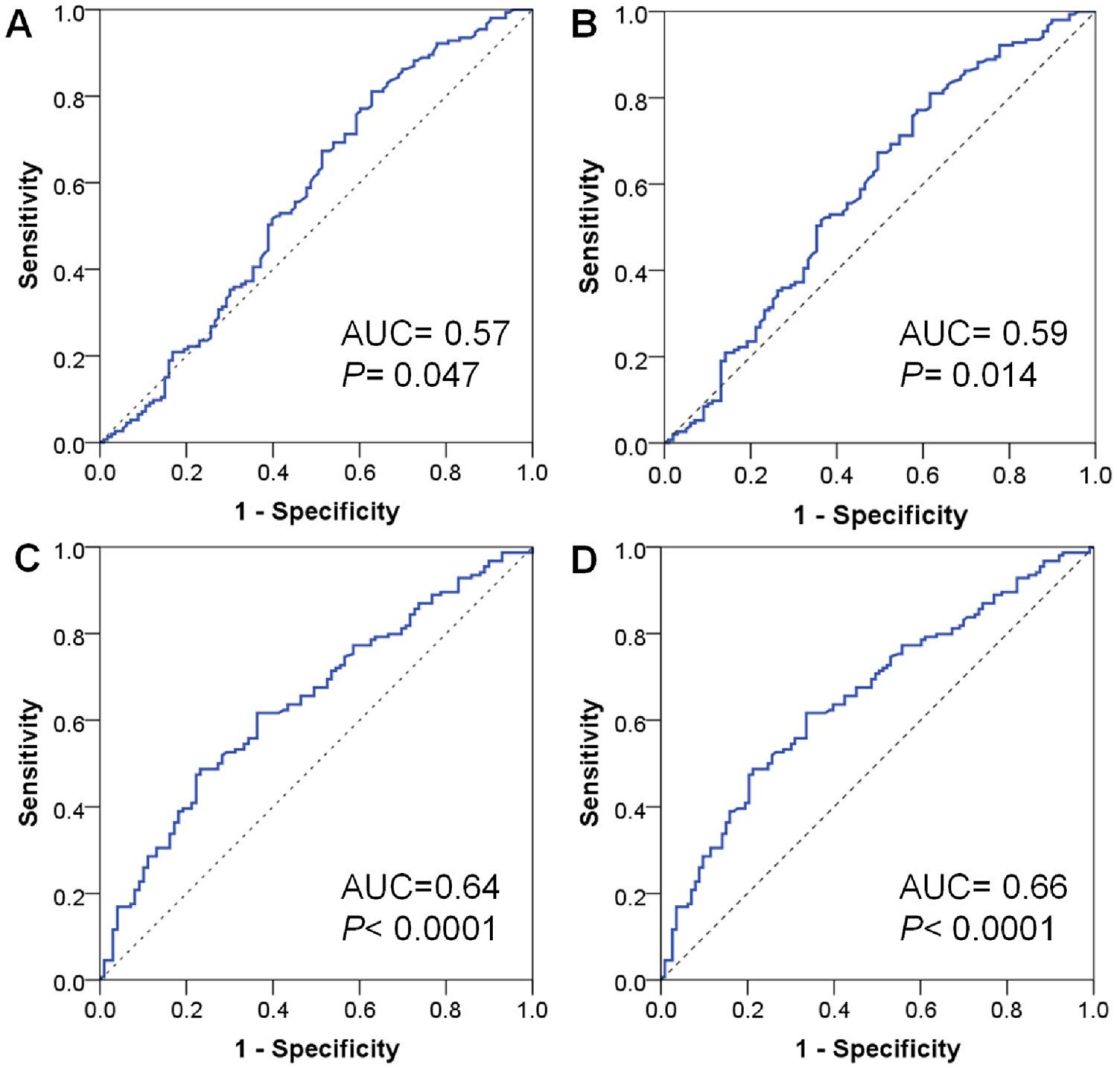
Prognostic performance of the CR1 and CR2 proteins for dengue progression. (**A** and **C**): ROC curve of CR1 and CR2 protein levels, respectively, in differentiating dengue with warning signs from dengue fever. (**B** and **D**): ROC curve of CR1 and CR2 protein levels, respectively, in differentiating dengue with warning signs and severe dengue from dengue fever.

We conducted an analysis of the prognostic performance in differentiating dengue patients at various disease stages. Our findings revealed that CR2 levels had the capability to discriminate between patients with DWS and those with SD from dengue fever patients (AUC = 0.66) with a designated cutoff value of 57.735 ng/ml (sensitivity of 61.7% and specificity of 66.4%). Conversely, CR1 levels had lower AUC values in distinguishing patients with DWS and SD patients from dengue fever patients (AUC = 0.59) (Fig. 4). These results underscore the potential of CR2 levels as an additional biomarker for prognosticating dengue disease severity.

### Association of CR1, CR2 gene polymorphisms and CR1, CR2 levels

**Figure 5:**
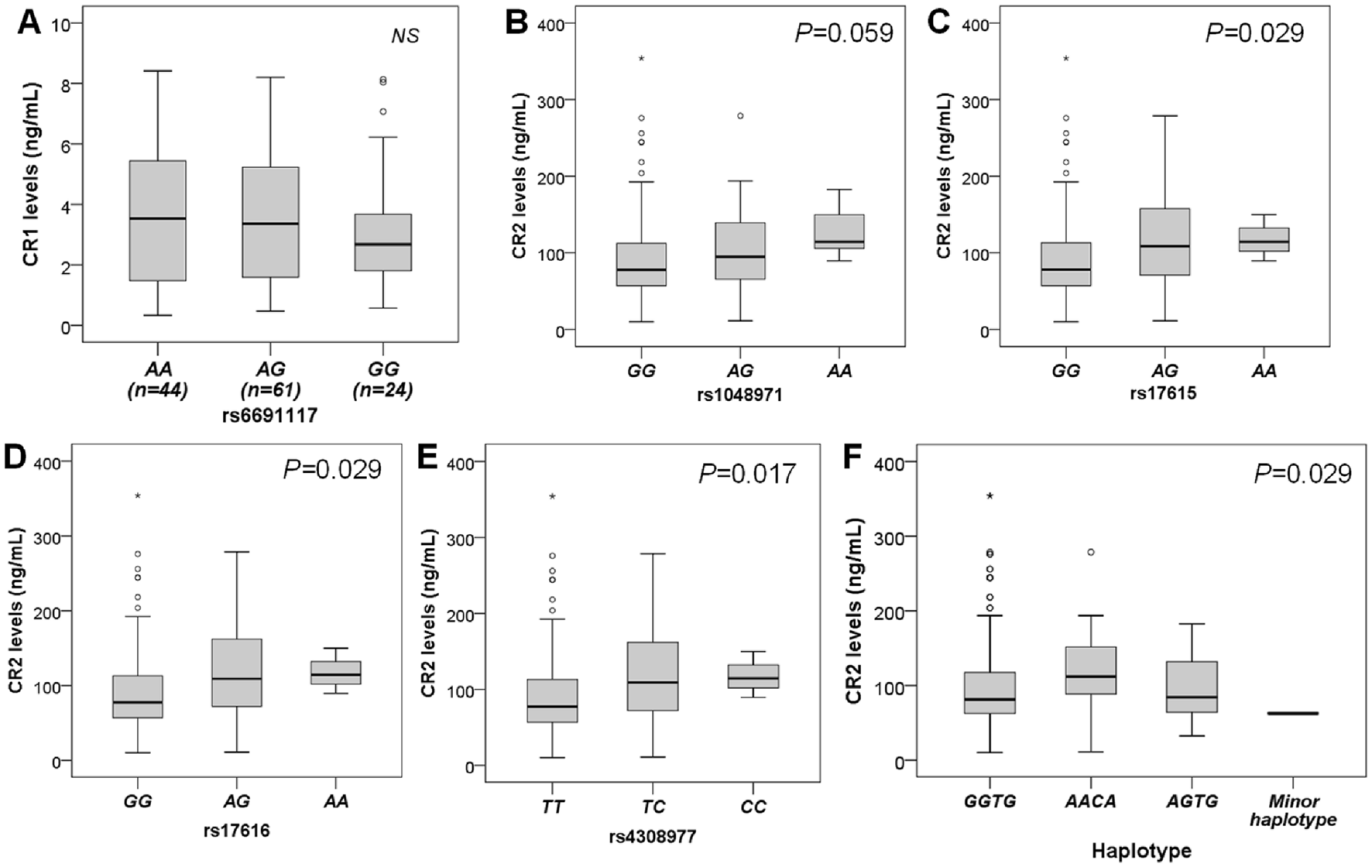
Distribution of CR1 and CR2 levels in healthy individuals with different *CR1* genotypes, *CR2* genotypes and haplotypes. (**A**): *CR1* SNP rs6691117; (**B**): CR2 SNP rs1048971; (**C**): *CR2* SNP rs17615; (**D**) *CR2* SNP rs17616; (**E**): *CR2* SNP rs4308977; (**F**): *CR2* haplotype; Box-plots present medians with 25 and 75 percentiles with whiskers to 10 and 90 percentiles, the dots and asterisks are the outliners; Comparison was performed using Kruskal–Wallis test. NS: not significant.

We explored the effect of *CR1* and *CR2* gene polymorphisms on the plasma CR1 and CR2 protein levels. In healthy controls, we observed that individuals with homozygous genotypes *rs1048971AA, rs17615AA, rs4308977CC,* and *rs17616AA* exhibited the highest CR2 levels, followed by heterozygous genotypes *rs1048971AG, rs17615AG, rs4308977TC,* and *rs17616AG* as compared to individuals with homozygous genotypes *rs1048971GG, rs17615GG, rs4308977TT,* and *rs17616GG*, respectively. We also observed a different distribution of CR2 levels among different *CR2* haplotypes. CR2 levels were highest in the *AACA* haplotype, followed by the *AGTG, GGTG,* and minor haplotypes. These results indicate that minor alleles of *CR2* SNPs contribute to increased CR2 levels (Fig. 5). However, we did not observe any significant difference in CR2 levels among different genotypes and haplotypes of *CR2* SNPs in dengue patients (Supplementary Fig. 1). These results imply that DENV infection influences plasma CR2 levels. In contrast, CR1 protein levels did not exhibit significant differences among different genotypes of *CR1* SNP rs6691117 either in healthy individuals or dengue patients (Fig. 5 and Supplementary Fig. 1), suggesting that *CR1* SNP rs6691117 may not contribute to regulating plasma CR1 levels.

## Discussion

The role of the complement activation pathway in DENV infection and the pathogenesis of dengue fever has been documented^21,22^. Although CR1 and CR2 have been associated with infections with several viruses such as HIV, HBV, and SARS-CoV-2^17–19^, their roles in the immune response to DENV infection and the progression of dengue fever have not been investigated, so far. The current study is the first to show an association between the *CR1* rs6691117A/G and *CR2* rs1048971G/A SNPs and the progression to severe dengue fever. Our findings reveal that the levels of CR1 and CR2 were modulated during the progression of dengue fever and were correlated with clinical parameters such as platelet counts and liver enzymes (ALT, AST). The reduction in CR2 levels was associated with the progression of dengue fever and with previous and current DENV infections. It thus may serve as an additional marker for the prognosis of DENV infection. In addition, the investigated *CR2* SNPs appear to affect plasma CR2 levels. Our results contribute to a deeper understanding of the roles of CR1 and CR2 during the immune response against DENV infection and the pathogenesis of dengue fever.

Several genetic loci associated with dengue fever have been identified in various studies, including *MICB* rs3132468 and *PLCE1* rs3765524^23^. In the complement system, polymorphisms in *MBL2* and *FCN2* genes have been associated with DENV infection in Brazilian and Vietnamese populations^24–27^. Regarding *CR1* polymorphisms, studies have shown the association of *CR1* gene polymorphisms with various diseases such as Alzheimer's disease, systemic lupus erythematosus (SLE), lower erythrocyte sedimentation rate, spontaneous idiopathic preterm birth, cancer, and infectious diseases^17,28–32^. Notably, a previous study suggests that the SNP rs6691117A/G is implicated in chronic Chagas disease, with the minor *rs6691117G* allele considered a risk factor^10^. Among SNPs in the exon 29 of the *CR1* gene (rs17259045, rs41274768, rs17047660, rs17047661, rs4844609, and rs6691117), which have been associated with pathogen detection, only SNP rs6691117A/G was observed in Vietnamese individuals, with the minor allele frequency of 36%^33^. In the current study, the minor allele *rs6691117G* appears to confer protection against dengue fever and progression to severe disease, although it has limited impact on plasma CR1 levels. Regarding *CR2* polymorphisms, a previous study identified *CR2* SNP rs3813946T/C as associated with increased susceptibility to nasopharyngeal cancer in the Cantonese ethnicity^15^. The rs311306 variant and haplotypes based on three SNPs, rs311306, rs17044576, and rs3767933, were associated with increased susceptibility to femoral head necrosis in the Korean population^16^. Moreover, haplotypes based on three *CR2* SNPs (rs3813946, rs1048971, rs17615) were associated with an increased risk of developing systemic lupus erythematosus (SLE), underscoring the potential role of CR2 in this disease^13^. The SNP rs3813946 was associated with increased susceptibility to HIV-1 infection after vaccination with recombinant glycoprotein 120 in the European population^19^. Gene polymorphisms influencing *CR2* expression and function have been associated with innate resistance to HIV infection^34^. Similarly, our results suggest that the *CR2* SNP *rs1048971G/A* is associated with an increased risk of progressing to severe dengue.

The role of CR1 in the activation of the alternative pathway (AP), mediated by CR2 in B cells, has been demonstrated to play an essential role in repairing C3b fragments. These fragments are produced by convertases and deposited at secondary acceptor sites on the surface of B cells, ultimately becoming a suitable ligand for CR2^35^. The function of CR1 is related to the activation of the complement system to protect against pathogens. Therefore, reduced CR1 levels and/or inactive CR1 may be associated with increased susceptibility to infections. Our results consistently indicate decreased CR1 levels in patient groups (DF and DWS), during the acute stages of DENV infection (T0 and T1), and a correlation between CR1 levels and platelet count. Interestingly, CR1 and CR2 levels were lower after 5 days of admission (T1) compared to the day of admission, but CR1 levels rapidly recovered within 8 days of follow-up, whereas CR2 levels did not exhibit the same recovery.

The *CR2* gene plays a pivotal role in innate immunity by inhibiting complement cascades of classical and alternative pathways and by regulating humoral immunity through activation and maturation of B cells^36,37^. The reduction in CR2 levels has been associated with the pathogenesis of rheumatoid arthritis^38^. Our findings indicate that CR2 levels decrease as dengue fever progresses through different stages. This phenomenon may be attributed to individuals with low CR2 levels having reduced activation of complement pathways, which are crucial for opsonizing and neutralizing DENV. Therefore, patients with lower CR2 levels tend to exhibit more severe clinical manifestations compared to those with higher CR2 levels. Notably, the decrease in CR2 levels in patients positive for anti-DENV IgG and IgM compared to those negative for anti-DENV IgG and IgM suggests that the secondary DENV infection led to more robust activation of the complement system than the primary DENV infection. When comparing CR2 levels between groups with and without signs of bleeding, higher CR2 levels were observed in the group without bleeding. The fact that CR2 can neutralize DENV indirectly by regulating the complement activation might explain this phenomenon. Therefore, higher CR2 levels can limit pathophysiological disorders during the infection, including signs of bleeding. In addition, CR2 levels were positively correlated with platelet count but inversely correlated with liver enzymes AST and ALT levels, further emphasizing CR2's role in responding to DENV infection and the progression of dengue fever. Furthermore, although the AUC value was on average, CR2 levels may be considered an additional prognostic marker for disease severity.

While CR1 levels were found to be correlated with CR2 levels in both patients and healthy controls, it became apparent that CR2 levels were lower in SD patients rather than CR1 levels. Furthermore, unlike CR2, CR1 levels were found to increase after 8 days of follow-up. These findings suggest that CR1 and CR2 may be involved in responding to DENV infection through different mechanisms. Consequently, further studies are necessary to elucidate the functional roles of CR1 and CR2 in DENV infection and the clinical progression to severe dengue.

In this study, minor alleles of *CR2* SNPs (*rs1048971A, rs17615A, rs4308977C,* and *rs17616A*) appear to be associated with higher CR2 levels in the control group. This finding further underscores the influence of *CR2* gene polymorphisms on plasma CR2 levels. However, a similar effect was not observed in dengue patients, probably due to the pathophysiological disorders during DENV infection and the influence of viral factors. The impact of gene polymorphisms on CR1 levels and function has been documented in various studies^10,28,29^. However, we could not show the significant influence of *CR1* SNP rs6691117A/G on CR1 levels in healthy controls and dengue patients. Therefore, the decrease in CR1 serum levels may be attributed to the pathological processes and anti-inflammatory effect of CR1.

Although this study represents the first attempt to elucidate the involvement of CR1 and CR2 in the progression of dengue fever, several limitations must be acknowledged. Notably, the number of patients with severe dengue was limited, potentially resulting in reduced statistical power when comparing genotype and allele frequencies of *CR1* and *CR2* SNPs. Nevertheless, it has provided the first hint for the association between *CR1* and *CR2* SNPs and the severity of dengue fever. A second limitation pertains to the number of *CR1* and *CR2* SNPs included in the investigation, although we selected *CR1* and *CR2* SNPs previously implicated in CR1 and CR2 functions and diseases.

In conclusion, the current study revealed that the *CR1* rs6691117A/G and *CR2* rs1048971G/A SNPs are associated with a progression to the severity of dengue fever. The levels of CR1 and CR2 were modulated during the clinical course of dengue fever, with the reduction in CR2 levels associated with disease severity and with previous or current DENV infections. Furthermore, CR2 levels were correlated with platelet counts, suggesting its potential as an additional prognostic marker for DENV infection. Further studies are warranted to elucidate the roles of CR1 and CR2 in the immune response against DENV infection and the progression of dengue fever.

### Supplementary Information

Supplementary Information.

## Data Availability

The datasets used and/or analyzed during the current study are available from the corresponding author upon reasonable request.
