# Supplementary material for: Complement receptor type 1 and 2 (CR1 and CR2) gene polymorphisms and plasma protein levels are associated with the Dengue disease severity
Source: Sci Rep. 2023 Oct 13;13:17377. doi: 10.1038/s41598-023-44512-w (PMC10575961; doi:10.1038/s41598-023-44512-w)
Supplement: Supplementary file 1 — Supplementary Information. [file 41598_2023_44512_MOESM1_ESM.docx]

**Supplementary data**

**Supplementary table 1: Genotype and allele distribution of *CR1* polymorphisms in dengue fever patients and healthy controls**

| **CR1 position** | **All Patients** | **DF** | **DWS** | **SD** | **Healthy Controls** |
| --- | --- | --- | --- | --- | --- |
| **rs6691117 (A/G)** | **n=267 (%)** | **n=154 (%)** | **n=99 (%)** | **n=14 (%)** | **n=133 (%)** |
| *AA* | 111 (41.6) | 63 (40.9) | 38 (38.4) | 10 (71.4) | 44 (33.1) |
| *GA* | 124 (46.4) | 67 (43.5) | 53 (53.5) | 4 (28.6) | 65 (48.9) |
| *GG* | 32 (12) | 24 (15.6) | 8 (8.1) | 0 (0) | 24 (18) |
| *P* for H&W equilibrium | 0.769 | 0.383 | 0.072 | 0.533 | 0.999 |
| *Allen* |  |  |  |  |  |
| *A* | 346 (64.8) | 193 (63.7) | 129 (65.2) | 24 (85.7) | 153 (57.5) |
| *G* | 188 (35.2) | 115 (37.3) | 69 (34.8) | 4 (14.3) | 113 (42.5) |
| *Dominant* |  |  |  |  |  |
| *AA* | 111 (41.6) | 63 (40.9) | 38 (38.4) | 10 (71.4) | 44 (33.1) |
| *GA+GG* | 156 (58.4) | 91 (59.1) | 61 (61.6) | 4 (28.6) | 89 (66.9) |
| *Resessive* |  |  |  |  |  |
| *AA+AG* | 235 (88) | 130 (84.4) | 91 (91.9) | 14 (100) | 109 (82) |
| *GG* | 32 (12) | 24 (15.6) | 8 (8.1) | 0 | 24 (18) |

**Supplementary table 2: Association of *CR1* polymorphisms with dengue fever in Vietnamese patients**

| ***CR1* Polymorphisms** | **All Patients vs. Healthy Controls** | | **DWS vs. Healthy Controls** | | **SD vs. Healthy Controls** | | **SD vs. DF** | | **SD vs. DWS** | |
| --- | --- | --- | --- | --- | --- | --- | --- | --- | --- | --- |
|  | **OR (95% CI)** | ***P* value** |  |  | **OR (95% CI)** | ***P* value** | **OR (95% CI)** | ***P* value** | **OR (95% CI)** | ***P* value** |
| **rs6691117 (A/G)** |  |  |  |  |  |  |  |  |  |  |
| *AA* |  |  |  |  |  |  |  |  |  |  |
| *GA* |  |  |  |  |  |  |  |  |  |  |
| *GG* |  |  |  |  |  |  |  |  |  |  |
| *Allen* |  |  |  |  |  |  |  |  |  |  |
| *A* |  | Reference |  | Reference |  | Reference |  | Reference |  | Reference |
| *G* | **0.74 (0.54-1.01)** | **0.045** |  | NS | **0.23 (0.06-0.69)** | **0.004** | **0.28 (0.07-0.85)** | **0.015** | **0.31 (0.08-0.96)** | **0.03** |
| *Dominant* |  |  |  |  |  |  |  |  |  |  |
| *AA* |  | Reference |  | Reference |  | Reference |  | Reference |  | Reference |
| *GA+GG* |  | NS |  | NS | **0.2 (0.04-0.74)** | **0.0046** | **0.28 (0.06-1.02)** | **0.027** | **0.25 (0.05-0.95)** | **0.019** |
| *Resessive* |  | NS |  | NS |  | NS |  | NS |  | NS |
| *AA+AG* |  | Reference |  | Reference |  | Reference |  | Reference |  | Reference |
| *GG* |  | NS | **0.4 (0.15-0.98)** | **0.029** |  | NA |  | NA |  | NA |

*P values were calculated by Chi-Squared test*

**Supplementary table 3: Genotype and allele distribution of *CR2* polymorphisms in dengue fever patients and healthy controls**

| **CR2 position** | **All Patients** | **DF** | **DWS** | **SD** | **Healthy Controls** |
| --- | --- | --- | --- | --- | --- |
| **rs1048971 (G/A)** | **n=267 (%)** | **n=154 (%)** | **n=99 (%)** | **n=14 (%)** | **n=133 (%)** |
| *GG* | 175 (65.5) | 102 (66.2) | 67 (67.7) | 6 (42.9) | 84 (63.2) |
| *GA* | 75 (28.1) | 43 (27.9) | 26 (26.3) | 6 (42.9) | 44 (33.1) |
| *AA* | 17 (6.4) | 9 (5.8) | 6 (6.1) | 2 (14.3) | 5 (3.8) |
| *P* for H&W equilibrium | 0.026 | 0.133 | 0.127 | 0.8 | 0.796 |
| *G* | 425 (79.6) | 247 (80.2) | 160 (80.1) | 18 (64.3) | 212 (79.7) |
| *A* | 109 (20.4) | 61 (19.8) | 38 (19.9) | 10 (35.7) | 54 (20.3) |
| **rs17615 (G/A)** | **n=267 (%)** | **n=154 (%)** | **n=99 (%)** | **n=14 (%)** | **n=133 (%)** |
| *GG* | 202 (75.7) | 120 (77.9) | 73 (73.7) | 9 (64.3) | 99 (74.4) |
| *GA* | 59 (22.1) | 31 (20.1) | 24 (24.2) | 4 (28.6) | 31 (23.3) |
| *AA* | 6 (2.2) | 3 (1.9) | 2 (2.0) | 1 (7.1) | 3 (2.3) |
| *P* for H&W equilibrium | 0.499 | 0.553 | 0.987 | 0.57 | 0.757 |
| *G* | 463 (86.7) | 271 (88) | 170 (85.9) | 22 (78.6) | 229 (86.1) |
| *A* | 71 (13.2) | 37 (12) | 28 (14.1) | 6 (21.1) | 37 (13.9) |
| **rs4308977 (T/C)** | **n=267 (%)** | **n=154 (%)** | **n=99 (%)** | **n=14 (%)** | **n=133 (%)** |
| *TT* | 202 (75.7) | 120 (77.9) | 73 (73.7) | 9 (64.3) | 100 (75.2) |
| *TC* | 59 (22.1) | 31 (20.1) | 24 (24.2) | 4 (28.6) | 30 (22.6) |
| *CC* | 6 (2.2) | 3 (1.9) | 2 (2.0) | 1 (7.1) | 3 (2.3) |
| *P* for H&W equilibrium | 0.499 | 0.553 | 0.987 | 0.57 | 0.676 |
| *T* | 463 (86.7) | 271 (88) | 170 (85.9) | 22 (78.6) | 230 (86.5) |
| *C* | 71 (13.2) | 37 (12) | 28 (14.1) | 6 (21.1) | 36 (13.5) |
| **rs17616 (G/A)** | **n=267 (%)** | **n=154 (%)** | **n=99 (%)** | **n=14 (%)** | **n=133 (%)** |
| GG | 201 (75.3) | 120 (77.9) | 72 (72.7) | 9 (64.3) | 99 (74.4) |
| GA | 59 (22.1) | 31 (20.1) | 24 (24.2) | 4 (28.6) | 31 (23.3) |
| AA | 7 (2.6) | 3 (1.9) | 3 (3.0) | 1 (7.1) | 3 (2.3) |
| *P* for H&W equilibrium | 0.297 | 0.553 | 0.57 | 0.57 | 0.757 |
| *G* | 461 (86.3) | 271 (88) | 168 (84.8) | 22 (78.6) | 229 (86.1) |
| *A* | 73 (13.7) | 37 (12) | 30 (15.2) | 6 (21.1) | 37 (13.9) |

**Supplementary table 4: Association of *CR2* polymorphisms with dengue fever in Vietnamese patients**

| ***CR2* Polymorphisms** | **SD vs. Healthy Controls** | | **DF vs. SD** | | **SD vs. DWS** | |
| --- | --- | --- | --- | --- | --- | --- |
|  | **OR (95% CI)** | ***P* value** | **OR (95% CI)** | ***P* value** | **OR (95% CI)** | ***P* value** |
| **rs1048971 (G/A)** |  |  |  |  |  |  |
| *GG* |  | Reference |  | Reference |  | Reference |
| *GA* |  | NS |  | NS |  | NS |
| *AA* | **5.6 (0.4-43.6)** | **0.042** |  | NS |  | NS |
| *G* |  | Reference |  | Reference |  | Reference |
| *A* | **2.2 (0.8-5.3)** | **0.06** | **2.2 (0.9-5.4)** | **0.048** | **2.3 (0.9-5.8)** | **0.045** |
| **rs17615 (G/A)** |  |  |  |  |  |  |
| *GG* |  | Reference |  | Reference |  | Reference |
| *GA* |  | NS |  | NS |  | NS |
| *AA* |  | NS |  | NS |  | NS |
| *G* |  | Reference |  | Reference |  | Reference |
| *A* |  | NS |  | NS |  | NS |
| **rs4308977 (T/C)** |  |  |  |  |  |  |
| *TT* |  | Reference |  | Reference |  | Reference |
| *TC* |  | NS |  | NS |  | NS |
| *CC* |  | NS |  | NS |  | NS |
| *T* |  | Reference |  | Reference |  | Reference |
| *C* |  | NS |  | NS |  | NS |
| **rs17616 (G/A)** |  |  |  |  |  |  |
| GG |  | Reference |  | Reference |  | Reference |
| GA |  | NS |  | NS |  | NS |
| AA |  | NS |  | NS |  | NS |
| *G* |  | Reference |  | Reference |  | Reference |
| *A* |  | NS |  | NS |  | NS |

*P values were calculated by Chi-Squared test*

**Supplementary table 5: Distribution of *CR2* haplotypes in dengue fever patients and healthy controls based on 4 SNPs**

| **CR2 Haplotype** | **All Patients** | **DF** | **DWS** | **SD** | **Controls** |
| --- | --- | --- | --- | --- | --- |
|  | **n = 492** | **n = 308** | **n = 198** | **N = 28** | **n = 266** |
| *GGTG* | 422 (79) | 247 (80.2) | 158 (79.8) | 17 (60.7) | 212 (79.7) |
| *AGTG* | 37 (6.9) | 24 (7.8) | 10 (5.1) | 3 (10.7) | 17 (6.4) |
| *AACA* | 71 (13.3) | 37 (12) | 28 (14.1) | 6 (21.4) | 36 (13.5) |
| *GGTA* | 2 (0.4) | 0 | 2 (1) | 0 | 0 |
| *AGTT* | 1 (0.2) | 0 | 0 | 1(3.6) | 0 |
| *GGTT* | 1 (0.2) | 0 | 0 | 1(3.6) | 0 |
| *AATA* | 0 | 0 | 0 | 0 | 1 (0.4) |

No dignificant differences between groups were observed in all comparison.

**Supplementary figure 1: Distribution of CR1 and CR2 levels in dengue patients with different *CR1* genotypes, *CR2* genotypes and haplotypes**

(A): CR1 SNP rs6691117; (B): CR2 SNP rs1048971; (C): CR2 SNP rs17615; (D) CR2 SNP rs17616; (E): CR2 SNP rs4308977; (F): CR2 haplotype; Comparison was performed using Kruskal-Wallis test. NS: not significant.
